# The Book World of Medicine and Science

**Published:** 1894-12-29

**Authors:** 


### The Book World of Medicine and Science.

**Prisoners and Paupers**: A Study of the Abnormal Increase of Criminals, and the Public Burden of Pauperism in the United States; the Causes and Remedies. By Henry M. Boies, M.A., Member of the Board of Public Charities, and of the Committee on Lunacy of the State of Pennsylvania; of the National Prison Association, &c.

This book is a serious and practical attempt to account for the enormous increase in crime and pauperism in America, with suggestions for their removal. While in England and Scotland crime is decreasing while population grows larger, America finds its criminal record far exceeding in proportion its absolute increase in numbers. The increase of the criminal class, says Mr. Boies, "is from 1 in 3,500 of our population in 1850 to 1 in 7,865 in 1890, or of 445 per cent.; while the population has increased but 170 per cent. in the same period." Among the causes to which this abnormal increase may be attributed Mr. Boies assigns the foremost place to unlimited immigration, and suggests severe restrictions on the admission of foreigners to the proud position of American citizens. Mr. Boies's suggestions are in the main sensible—that emigrants should, before leaving home, be forced to provide themselves with certificates, issued by the nearest U.S. Consul, that they are sound physically and mentally, and have a reasonable prospect of not becoming paupers. But it is not easy to see how the Consul in a large town is to obtain satisfactory information on these points, especially the last, which would probably result in some such narrow and futile regulation as that he should possess a certain sum of money in hand after his passage is paid. Mr. Boies is also a thorough-going Protectionist, and would forbid the importation of "low-grade contract labour," as tending to lower the wages of the native worker. But Mr. Boies must be aware that the existing penalty attached to the importation of labour simply causes endless lying and evasion at every American port, while in so far as it is successful it causes much useful and necessary work to be left undone, or left to casuals who cannot do even low grade work well, because it is beneath the ambition, social or pecuniary, of the American citizen. Mr. Boies himself complains that country life is made unattractive and farming unprofitable through the badness of the roads outside the immediate vicinity of the great cities. Surely "low grade contract labour" might be brought to bear on this. He laments, too, that emigrants now seek America more to share American wealth than to enjoy American freedom. This is true; but we think it is those who come for the sake of the liberty of the West who are likely to make the worst citizens. A man who comes to any country with the fixed purpose of making a living in it is not likely to end as either criminal or pauper.

Other causes of crime and pauperism Mr. Boies finds in the neglect and cruelty which are still meted out to the negro race in the South; to alcohol; and to a system of gaols and penitentiaries which seems specially designed for social corruption. It seems incredible that in America, at this period of the nineteenth century, hardened criminals, children who have been guilty of mere mischief or petty larceny, detained witnesses, and persons who have not been tried and may be guiltless, should all be herded together. We quote from Mr. Boies's personal investigation of the inmates of the gaol at Sunbury, "reckoned one of the best in the State" (Pennsylvania):—"Among them were two bright, nice-looking boys, one thirteen and the other fourteen years old, who had been incarcerated already two months, and would have to remain two months longer before trial. They were accused of stealing four bottles of ginger beer! Another boy of sixteen years was awaiting trial for attempted rape—a depraved and vicious-looking wretch." How would the first two come out after so long association with the third?

With regard to alcohol, Mr. Boies sensibly suggests that it should at least "pay its own way"—that it should be taxed enough to pay for the building and maintenance of the gaols, poor-houses, and asylu ms which it helps to fill.

With regard to the reduction and prevention of crime, however, Mr. Boies is not afraid to recommend the most drastic measures. Taking crime, whether directly due to alcoholism or not, as a disease, he would decree a life sentence wherever an evident criminal diathesis exists—say at the third conviction. But he would go further than this. He would prevent the development of those criminal families whose growth we are only now beginning to note. This is a very serious step. To take away even life itself may be regarded as a less interference with the law of Nature than to take away the power of giving life, though it is true that Mr. Boies justifies his suggestion on the ground that as we interfere with Nature's method of killing the unfit by starvation and disease, we ought, in compensation, to go further and interfere with the reproduction and multiplication of the unfit. He would, indeed, have all marriage severely controlled, with a view to the improvement of the race. His advice on this point we regard as impracticable, and likely, indeed, to lead to evils as serious as those he desires to prevent. It is a good thing to bring up young persons with a high ideal—moral, physical, and intellectual—of what husband and wife should be; to let them realise clearly the responsibilities and consequences of marriage; and to give them as large a circle as possible to select from; but beyond such indirect guidance it is not safe to go, and further interference would probably lead to rebellion and demoralization.

